# Local Injection of Allogeneic Stem Cells from Apical Papilla Enhanced Periodontal Tissue Regeneration in Minipig Model of Periodontitis

**DOI:** 10.1155/2018/3960798

**Published:** 2018-07-12

**Authors:** Guoqing Li, Nannan Han, Xiuli Zhang, Haoqing Yang, Yangyang Cao, Songlin Wang, Zhipeng Fan

**Affiliations:** ^1^Laboratory of Molecular Signaling and Stem Cells Therapy, Beijing Key Laboratory of Tooth Regeneration and Function Reconstruction, Capital Medical University School of Stomatology, No. 4 Tiantanxili, Dongcheng District, Beijing 100050, China; ^2^Molecular Laboratory for Gene Therapy and Tooth Regeneration, Beijing Key Laboratory of Tooth Regeneration and Function Reconstruction, Capital Medical University School of Stomatology, Beijing 100050, China; ^3^Department of Periodontology, Capital Medical University School of Stomatology, Beijing 100050, China; ^4^Department of Biochemistry and Molecular Biology, Capital Medical University School of Basic Medical Sciences, No.10 Xitoutiao Youanmen, Fengtai District, Beijing 100069, China

## Abstract

**Background:**

Discovering suitable seeding cells and simple application technique will be beneficial for MSC-mediated treatment of periodontitis. Stem cells from apical papilla (SCAPs) might be the candidate seeding cell for the periodontal tissues regeneration based on their origin and characters. In this research, we investigated the effect of SCAPs on periodontal tissue regeneration in swine by local injection.

**Methods:**

We established experimental periodontitis model in miniature pigs and then treated them with SCAPs by local injection. Clinical assessments, computed tomography (CT) scanning, histologic examination, and quantitative measurements were used to evaluate the effect of periodontal tissues regeneration.

**Results:**

At 12 weeks after injection, clinical assessments showed that probing depth, gingival recession, and attachment loss values were 5.44±0.77 mm versus 7.33±1.0 mm (p<0.01), 2.33±0.33 mm versus 2.11±0.69 mm (p>0.05), and 7.78±0.84 mm versus 9.44±1.07 mm (p<0.01) in SCAPs group and 0.9% NaCl group, respectively. CT scan results showed a significant increase of 12.86 mm^3^ alveolar bone regeneration in SCAPs group compared with 0.9% NaCl group. In addition, histopathology results demonstrated remarkable regeneration in SCAPs group, whereas regeneration of periodontal tissue was hardly found in 0.9% NaCl group.

**Conclusion:**

Local injection of SCAPs could effectively restore tissue defects brought about by periodontitis in the swine model. Thus, SCAPs, as an easily accessible dental-deriving stem cell, may serve as an alternative application for periodontitis treatment.

## 1. Introduction

Periodontitis is a wide-spread chronic infectious disease that can destruct teeth supporting tissues, associates with many other diseases, and is the main cause of tooth loss of adults [1–5]. While the effects of restoring hard and soft periodontal tissues by conventional treatments remain unsatisfactory [6, 7]. Thus far, appropriate methods are necessary for ideal regeneration of periodontal tissues. Recent years, cell-based therapy using mesenchymal stem cells (MSCs) is becoming a promising alternative strategy [8–12]. Previous studies focus on two types of MSCs on periodontal tissue regeneration including dental-derived MSCs such as PDLSCs (periodontal ligament stem cells), DPSCs (dental pulp stem cells), and non-dental-derived MSCs like BMSCs (bone marrow mesenchymal stem cells) [13–17]. They both have the potential to promote periodontal tissues regeneration, while dental tissue-derived MSCs have the peculiarities of their differentiation, proliferation, and immunosuppress capacities which may apply better effect on oral tissue regeneration. PDLSCs and DPSCs have been considered promising seed cells for periodontal tissue regeneration [16, 17]. Comparison showed that PDLSCs and DPSCs expressed similar MSC markers with different levels. DPSCs had higher doubling rate and telomerase activity than PDLSCs [18, 19], while results of alkaline phosphatase (ALP) activity and mineralization studies suggested stronger osteogenic differentiation potential in PDLSCs than DPSCs [20, 21]. SCAPs are another kind of dental-derived MSCs from apical papilla of teeth with incompletely developed station, which is capable of self-renewal and differentiating into different types of cells [22, 23]. Researchers have shown that SCAPs, which contribute to the formation of odontoblast-like cells, are capable of regenerate pulp-like tissue in emptied root canal space [24, 25]. Also, higher proliferation, greater mineralization capacity and stronger osteogenic differentiation potential were found in SCAPs compared to that of PDLSCs and DPSCs, which makes SCAPs a candidate alternative seed cell for bone and dental tissue regeneration [24, 26–28].

Besides, previous researches on periodontal regeneration mainly relied on scaffold-based approaches, but problems such as host rejection, complication of transplantation, different degradation, and cell proliferation rate are the main concerns. Thus, non-scaffold tissue engineering strategy including cell injection and cell sheet has been of great interests to researchers these years. Cell sheet may be beneficial for stem cell-based tissue regeneration because of its mimicking cellular microenvironments and maintenance of endogenous extracellular matrix (ECM). However, periodontal flap operation was needed during cell sheet transplantation which is traumatic for patients. Cell injection has been used as therapy to many diseases and might also be a common approach to treat periodontitis. And the main superiority of cell injection is to provide a minimally invasive process. In our previous work, local injection of BMMSCs was used to treat periodontitis in rat and has good effect [8].

Nevertheless, study about SCAPs on periodontitis tissue regeneration is rarely found; we considered it a promising cell source by using the simple application technique on periodontitis treatment. In the present study, we aim to figure out whether SCAPs are a candidate seed cell for periodontal tissue regeneration by using the method of local injection. Periodontitis model was generated in minipigs and SCAPs were used by local application as the treatment. Clinical assessments, CT scans, and histopathology results showed that SCAPs were capable of serving as an appropriate alternative for periodontal regeneration.

## 2. Material and Methods

### 2.1. Cell Isolation and Culture

This study was approved by Ethical Committee of Beijing Stomatological Hospital (Review No. 2011-02) before the research started. Written informed consent was obtained from each patient before human impacted third molar with immature roots were collected under the guidelines of Beijing Stomatological Hospital, Capital Medical University. Wisdom teeth extracted from patients were stored in phosphate buffered saline (PBS) or serum-free cell culture medium immediately. Then 75% ethanol was used to disinfect the teeth and washed with PBS for several times. Apical papilla was separated gently from the tip of the root and cut into small pieces. Subsequently, the tissues were digested in a mix solution of 3 mg/mL collagenase type I (Worthington Biochemical Corp., Lakewood, NJ, USA) and 4 mg/mL dispase (Roche Diagnostics Corp., Indianapolis, IN, USA) for about 1 h at 37°C and then passed the digested tissues through a 70-*μ*m strainer (Falcon, BD Labware USA) to obtain isolated cell suspensions. SCAPs were grown in a humidified incubator under 5% CO_2_ at 37°C in DMEM alpha modified Eagle's medium (Invitrogen, Carlsbad, CA, USA), supplemented with 15% fetal bovine serum (FBS; Invitrogen), 2mmol/l glutamine, 100 U/ml penicillin, and 100 *μ*g/ml streptomycin (Invitrogen). The culture medium was changed every 3 days. SCAPs were identified [29, 30] and cells at passages 3-5 were used in subsequent experiments.

### 2.2. Animals

Six inbred male minipigs, 18 months old and weighing 55-60kg, were obtained from the Institute of Animal Science of the Chinese Agriculture University (Beijing, China) and were housed under conventional conditions, with free access to water and a regular supply of soft food diet. The study protocol was approved by the Animal Care and Use Committee of Capital Medical University. Prior to surgery, the minipigs were clinically assessed and then anesthetized with a combination of ketamine chloride (6 mg/kg) and xylazine (0.6 mg/kg).

### 2.3. MSC-Mediated Treatment for Swine Periodontitis

12 periodontitis defects were generated in 6 minipigs. Defects with a size of 3 mm (width) × 5 mm (depth) × 7 mm (length) in the mesial region of the bilateral mandibular first molars were created by removing alveolar bone and cement with a 4-0 silk ligament sutured around the cervical portion after the operation. On the root surface, notch-shaped marks were made at the floor of the defects. Then the minipigs received injections at three sites surrounding each defect: the mesial, the distal, and the middle of the molar four weeks after surgery. Animals were randomly assigned to each group with split mouth method (6 minipigs for each group), injected with 0.9% NaCl (0.9% NaCl group) and SCAPs+ 0.9% NaCl (SCAPs group). In each site around the defect, SCAPs with approximately 2 × 10^6^ SCAPs in 0.2 ml 0.9% NaCl were injected subperiosteally with the needle entered from the mucosa straight to the surface of bone.

### 2.4. Clinical Assessments and CT Scanning of Periodontal Tissue Regeneration

Clinical assessments, including probing depth (PD), gingival recession (GR), and attachment loss (AL), were measured on each experimental tooth right before the generation of periodontitis models, before the treatment, and 12 weeks after transplantation. The regeneration of newborn bone was analyzed based on three-dimensional reconstructive CT scan (Siemens, Berlin, Germany) using Geomagic 12 by fitting three-dimensional reconstruction images at the indicated time points before the treatment and 12 weeks after transplantation; then a growth volume of newly regenerated tissue can be obtained and calculated. The CT scanning length was 0.62 mm.

### 2.5. Histologic Assessments

12 weeks after transplantation, samples were harvested, fixed with 4% formaldehyde (PFA, PH=7.4-7.6), decalcified with 10% ethylene diamine tetraacetic acid (EDTA, pH 7.4-7.6), and then embedded in paraffin. Sections (5 *μ*m) were stained with hematoxylin and eosin (H&E). The volume of new cementum was analyzed using image-Pro 1.49v (National Institutes of Health, Bethesda, MD, USA).

### 2.6. Statistics

All statistical calculations were performed using SPSS10 statistical software. Student's* t*-test was utilized to determine statistical significance, with a p value of ≤.05 considered significant.

## 3. Results

### 3.1. Improved Gingival Status and Clinical Assessments in SCAPs Group Compared to 0.9% NaCl Group

**Figure 1:**
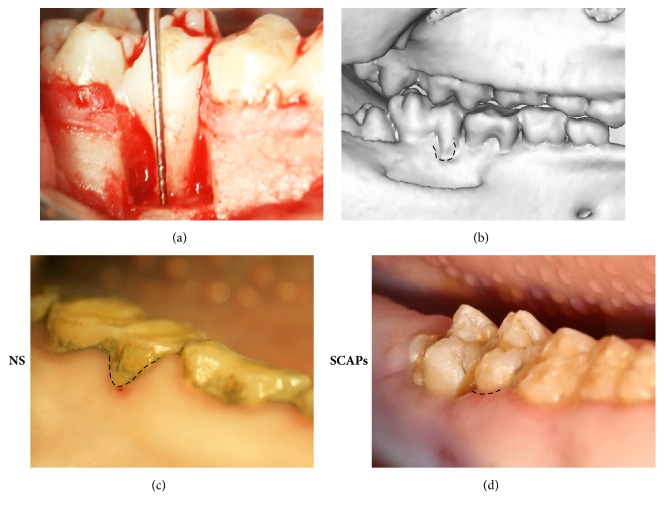
*Local application of SCAPs for periodontitis treatment in a minipig periodontal model*. (a-b) Bone defects of 3 mm × 5 mm × 7 mm were generated in the mesial region of the bilateral mandibular first molars (a). Three-dimensional CT images showed obvious bone defect in experimental region (b). (c-d) Intraoral manifestations demonstrating tissue regeneration in 0.9% NaCl group (c) and SCAPs group 12 weeks after the treatment (d).

To investigate the effect of SCAPs for tissue regeneration, experimental periodontitis model was created in 6 minipigs with 12 defects (Figure 1(a)); CT scans showed successful model was established after 4 weeks (Figure 1(b)). 12 weeks after transplantation, intraoral photographs showed that gingival appearance is better in SCAPs group than that in 0.9% NaCl group (Figures 1(c) and 1(d)).

**Figure 2:**
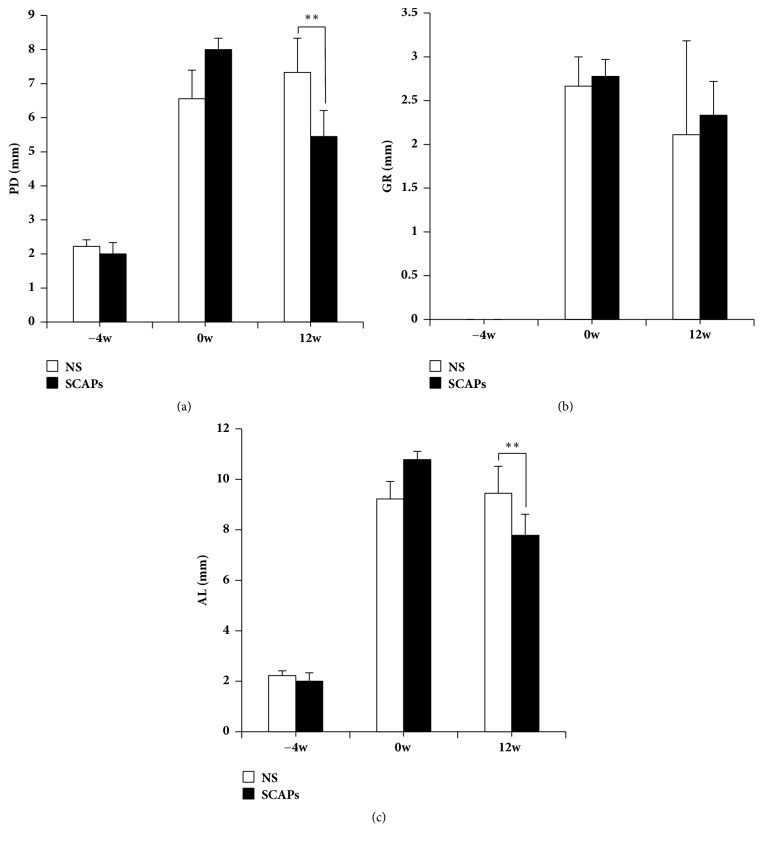
*Clinical assessments of the periodontal situation*. (a–c) 12 weeks after transplantation, the values of PD and AL were significantly improved in the SCAPs group compared to that of the 0.9% NaCl group, while GR values had no significance. Bars and vertical lines: mean±standard deviation. Student's* t*-test was applied to test statistical significance. *∗∗*p<0.01. PD, probing depth; AL, attachment loss; GR, gingival recession.

Animals were then separated into two groups by split mouth method by injecting with SCAPs+0.9% NaCl or 0.9% NaCl, respectively. 12 weeks after treatment, PD values were 7.33±1.0mm in the 0.9% NaCl group and 5.44±0.77mm in the SCAPs group (p<0.01) (Figure 2(a)). GR values were 2.11±0.69 mm in the 0.9% NaCl group and 2.33±0.33mm in the SCAPs group (p>0.05), which had no significant difference (Figure 2(b)). As for AL, the values were 9.44±1.07mm in the 0.9% NaCl group and 7.78±0.84 mm in the SCAPs group (p<0.01) (Figure 2(c)).

These results revealed that injection of SCAPs can promote the recovery of gingival and led to improvement of PD and AL.

### 3.2. CT Scan Results Showed More Bone Regeneration after SCAPs Application

**Figure 3:**
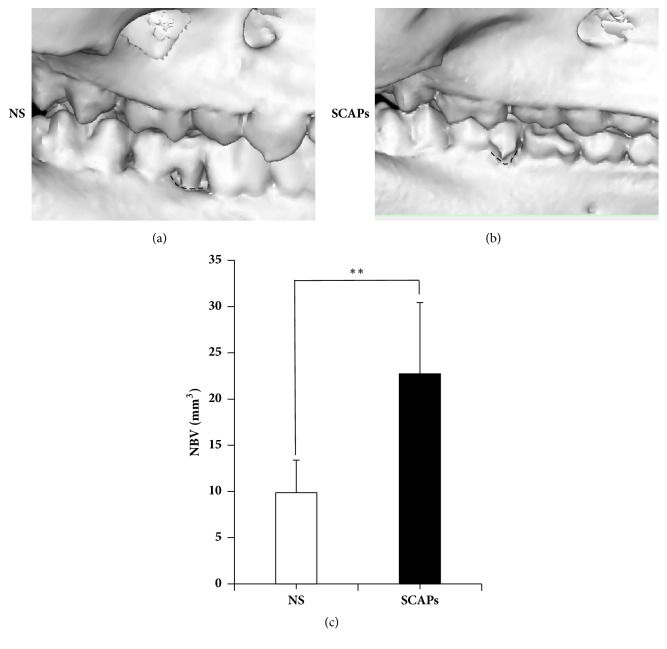
*CT scan showed superior regenerative periodontal tissue in SCAPs group compared with 0.9% NaCl group*. (a, b) Three-dimensional CT images revealed limited bone formation in 0.9% NaCl group 12 weeks after transplantation (a). SCAPs-mediated nearly complete alveolar bone regeneration (b). Analysis of CT results showed that the efficiency of newborn bone in the SCAPs group was significantly better than the 0.9% NaCl group (c). Student's* t*-test was performed to determine statistical significance (*∗∗*p<0.01).

12 weeks after treatment, three-dimensional reconstruction results showed much more alveolar bone regeneration in SCAPs group (Figures 3(a) and 3(b)). Computed tomography imaging showed higher alveolar in SCAPs group (Supplementary Fig1A, B). Quantitative analysis showed that the volume of newborn bone in the SCAPs group is 22.73±4.94 mm^3^ versus 9.87±3.53 mm^3^ in 0.9% NaCl group (Figure 3(c), p<0.01), demonstrating that SCAPs enhanced bone formation in minipigs periodontitis defects model.

### 3.3. Histopathologic Assessment Demonstrated That SCAPs Enhanced Periodontal Tissues Regeneration

**Figure 4:**
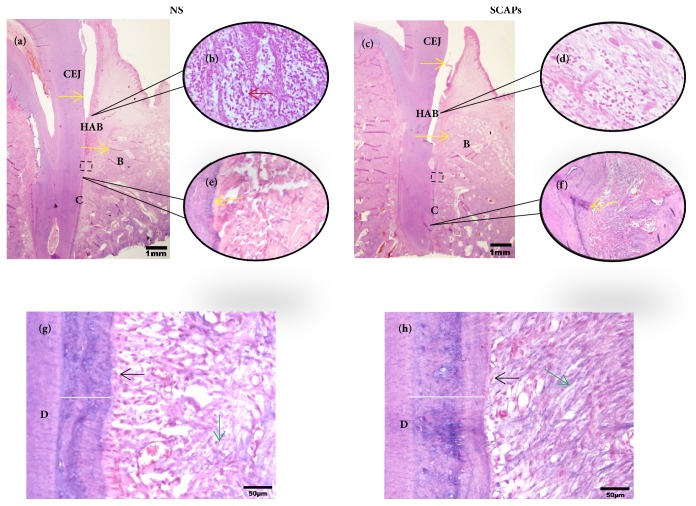
*Whole view of histopathologic assessment for periodontal tissue regeneration by HE*. HE staining showing new periodontal tissue regeneration in the periodontal defect area in 0.9% NaCl group (a, b, e, and g) and SCAPs group (c, d, f, and h). (b) Enlarged view of sulcular epithelium of 0.9% NaCl group; (d) enlarged view of sulcular epithelium of SCAPs group; (e) and (f) enlarged view shows notch-shaped marks (yellow arrow) made in 0.9% NaCl group and SCAPs group. (g, h) Enlarged view of periodontal tissues in 0.9% NaCl group and SCAPs group (black rectangular area in a and b). Red arrow, inflammatory cells; green arrow, Sharpey's fibers; black arrow, cementoblast; white bar, the width of new cementum. Bar: =1mm (a, b), 100 *μ*m (e, f), and 50*μ*m (g, h). D, dentin; C, cementum; B, bone; CEJ, cemento-enamel junction; HAB, height of alveolar bone.

**Figure 5:**
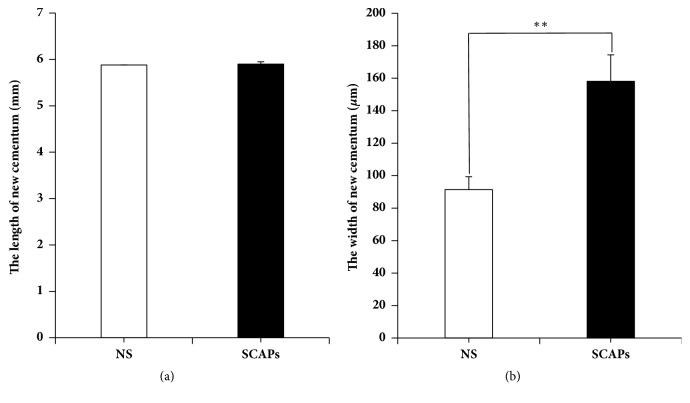
*Quantitative analysis of new cementum showed more regeneration of new cementum in SCAPs group*. (a, b) Quantitative analysis of HE staining showing new cementum regenerated in SCAPs group compared with 0.9% NaCl group. Student's* t*-test was performed to determine statistical significance. Error bars represent SD (n = 6). *∗∗*p ≤ 0.01.

Histopathologic assessment showed that typical periodontitis features still existed in 0.9% NaCl group, including deep periodontal pocket, abundant inflammatory cell infiltration in sulcular epithelium, and lack of periodontal ligament and the typical structure of Sharpey's fibers (Figures 4(a), 4(b), 4(e), and 4(g)). In contrast, typical structure of Sharpey's fibers and a lot of periodontal ligament were regenerated in SCAPs group. Moreover, few inflammatory cells and newly formed Sharpey's fibers could also be observed in SCAPs group. There was thicker and more mature regenerated cementum in SCAPs group compared to that in 0.9% NaCl group (Figures 4(c), 4(d), 4(f), and 4(h)). Although the length of new generated cementum showed no difference (Figure 5(a)), the width is 91.4±7.9 *μ*m in 0.9% NaCl group versus 158.1±16.3 *μ*m in the SCAPs group (Figure 5(b), p<0.01), which indicated more new cementum regenerated in the SCAPs group.

## 4. Discussion

In this research, we investigated the effect of SCAPs-mediated therapy for periodontitis in minipigs. Periodontitis model was established in minipigs and local injection of SCAPs was used as the treatment. 12 weeks after the treatment, clinical assignment, CT scan, HE staining, and quantitative analysis demonstrated superior regenerative effect of periodontal tissue in SCAPs group, indicating that SCAPs, as a dental tissue-derived MSCs, might serve as suitable alternative cell source for periodontal tissue engineering.

Periodontitis leads to destruction of periodontal supported tissues such as alveolar bone, cementum, periodontal ligament, and gingiva. The purpose of treatment is to cease the periodontitis process, regenerate tissues, and restore periodontal function [28]. Researches showed MSCs-mediated therapy a promising treatment, in which appropriate seed cell is the key process, so broaden and better alternative of the cell source is still in great need.

Dental-derived MSCs based therapy for periodontitis has approved certain achievements. Both allogeneic and autologous PDLSCs are believed to have great therapeutic effect on regeneration of soft and hard periodontal tissues in animal models [16, 31], but the source of PDLSCs is limited. SCAPs can be obtained from unmatured extracted wisdom teeth. Apical papilla is the precursor of root, which is composed of cells with more undifferentiated ability than DPSCs and PDLSCs. SCAPs express MSCs markers and can differentiate into different cell types such as neural cells, adipocytes, odontogenic cells, and formatting vascularized pulp-like tissue in vivo [22, 32, 33]. On treatment of periodontitis, SCAPs showed their superiority compared with other MSCs such as non-dental-derived MSCs like BMMSCs and dental-derived MSCs including PDLSCs and DPSCs. As to BMMSCs, SCAPs had similar potential in osteo/dentinogenic differentiation but higher proliferation rate, and they are easier to isolated [20]. In comparison with PDLSCs and DPSCs, SCAPs are superior with their MSCs capacity on population doubling rate and differentiation ability [24, 26]. Otherwise, SCAPs have been confirmed to have the ability to suppress the immune reaction through suppressing T cell proliferation which may help broaden the use of allogenic SCAPs transplantation by decreasing the immunoreaction [34]. With the discovery of key factors that maintain the function of SCAPs [35], strategies such as gene modification and the combined use of growth factor may be provided as effective attempts, while further investigation of the detailed mechanism about how SCAPs mediate periodontal tissue regenerative in periodontitis model still needs to be done.

## 5. Conclusion

Our data demonstrated that local injection of SCAPs can enhance the regeneration of periodontal tissue, which may serve as a promising therapy for periodontal tissue regeneration in the future.

## Data Availability

The data used to support the findings of this study are included within the article.

## Supplementary Materials

Supplementary Materials Supplementary material contains computed tomography imaging of buccal lingual view in 0.9% NaCl group and SCAPs group (Supplementary Figure 1).
