# Supplementary material for: Local Injection of Allogeneic Stem Cells from Apical Papilla Enhanced Periodontal Tissue Regeneration in Minipig Model of Periodontitis
Source: Biomed Res Int. 2018 Jul 12;2018:3960798. doi: 10.1155/2018/3960798 (PMC6077668; doi:10.1155/2018/3960798)
Supplement: Supplementary Materials — Supplementary material contains computed tomography imaging of buccal lingual view in 0.9% NaCl group and SCAPs group (Supplementary Figure 1). [file 3960798.f1.docx]

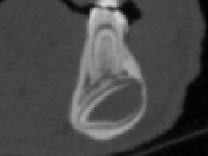

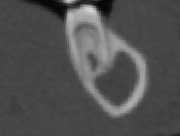


**NS**

**SCAPs**

**A**

**B**

**Supplementary Figure 1** Computed tomography imaging of buccal lingual view in 0.9% NaCl group (A) and SCAPs group (B). The white arrow showed the height of alveolar.
